# CONTEMPORARY EARLY RETIREMENT FACTORS AND STRATEGIES TO ENCOURAGE AND ENABLE LONGER WORKING LIVES

**DOI:** 10.1093/geroni/igz038.2400

**Published:** 2019-11-08

**Authors:** Donna M WILSON

**Affiliations:** University of Alberta, Edmonton, Alberta, Canada

## Abstract

Accelerating population aging is raising concern in many countries now about the availability of workers for essential work roles and responsibilities. A scoping research literature review was done to identify factors currently associated with early retirement and contemporary strategies to encourage and support longer working lives. Among 53 relevant articles, seven early retirement factors were revealed: Ill health, good health, workplace issues, the work itself, ageism, social norms, and having achieved personal financial or pension requirement criteria. Six solutions, none of which had been proven effective, were identified: Occupational health programs, workplace enhancements, work adjustments, addressing ageism, changing social norms, and pension changes. The evidence base on early retirement prevention is not strong, with qualitative research studies needed to gain a more in-depth understanding of early retirement influences and also mixed-methods studies needed to test early retirement prevention solutions for their effects, and particularly in the healthcare sector as healthcare needs typically rise with advanced ageing. Until more evidence is available, every healthcare and other organization should perform an early retirement risk assessment and identify current versus needed policies and programs to encourage and to enable more middle-aged and older people to work longer in life.

